# Characterizing the Efficacy of a Film-Forming Antitranspirant on Raspberry Foliar and Fruit Transpiration

**DOI:** 10.3390/biology9090255

**Published:** 2020-08-31

**Authors:** Francesca J. Moroni, Pedro J. Gascon-Aldana, Suzy Y. Rogiers

**Affiliations:** 1 National Wine and Grape Industry Centre, Charles Sturt University, Wagga Wagga, NSW 2678, Australia; fmoroni@csu.edu.au (F.J.M.); pedrogasconj@gmail.com (P.J.G.-A.); 2 NSW Department of Primary Industries, Wagga Wagga, NSW 2678, Australia

**Keywords:** climate change, drought, pinolene, *Rubus*, stomatal conductance, transpiration efficiency

## Abstract

The film-forming antitranspirant, di-1-*p*-menthene, is able to reduce transpiration in a number of crops, potentially resulting in water savings and improved productivity. The success of the response is, however, dependent on genotype and environmental factors. We aimed to assess the efficacy of this natural terpene polymer on red raspberry (*Rubus idaeus*, L.) cv. Tulameen leaf water-use efficiency across a 25–40 °C temperature range under controlled conditions. The film reduced transpiration (*E*) and was most effective when applied to the lower leaf surface. Leaf net assimilation (*A*) and stomatal conductance (*g*) were also curtailed after the application of di-1-*p*-menthene, and as a consequence intrinsic transpiration efficiency (*A/g*) and instantaneous transpiration efficiency (ratio of net carbon fixation to water loss, *A/E*) did not improve. At 40 °C, gas exchange of both treated and untreated leaves was minimal due to stomatal closure. The antitranspirant was effective at reducing water loss from berries, but only at the immature stages when transpiration rates were naturally high. Further studies are required to determine if the antitranspirant, di-1-*p*-menthene, will offer protection against dehydration across a range of temperatures and if productivity and berry composition will benefit.

## 1. Introduction

Raspberries are predominantly grown in the temperate regions of central and northern Europe, but production is expanding to warmer regions, including those of Australia. Climate change is resulting in warmer temperatures and unpredictable and severe weather events, putting temperate horticulture species such as *Rubus* at risk. Raspberries are susceptible to climate change because they are not well adapted to warm temperatures and drought, preferring cooler summers with moderate rainfall [1]. Raspberry is shallow-rooted, and prolonged water stress results in altered phenology [2], changes in nutrient and carbon partitioning [3], and increased susceptibility to diseases and insects [1]. A water deficit as a result of an unreliable water supply can reduce yield in the current [4] and the following season [2].

Alongside changes in growth and phenology [5,6], leaf photosynthesis and stomatal conductance are curtailed when raspberry plants are exposed to higher temperatures [7,8,9] and water stress [2,10,11,12]. Since stomatal closure restricts transpiration, it is an effective water saving mechanism for the short-term. However raspberry plants are unable to sustain moderate to severe long-term water stress because carbon accumulation can be severely hampered [2]. Water stress in young developing orchards can result in stunted growth and poor block establishment. Many Australian horticultural production regions have been experiencing more frequent and more severe droughts, and interest in antitranspirants is growing. Considering that our precious water resources are limited, finding methods to improve plant performance under drought is a common goal across many agricultural industries. Drought tolerance or avoidance will not only expand the geographical boundaries for production but are also relevant to controlled environment conditions such as glasshouses and tunnels.

Aside from the implementation of deficit irrigation strategies (concomitant with vigilant monitoring of plant water status), improvements in the efficiency of water-use can be achieved through the foliar application of antitranspirants [13,14]. These antitranspirants can be natural or synthetic, and may form a protective barrier [15], reflect sunlight and lower leaf temperature [16] or induce guard cell closure [17]. Antitranspirants have had positive effects on plant performance in strawberry, gooseberry, kiwi, tomato, apple, banana, mango and other fruits when exposed to drought [14]). Di-1-*p*-menthene (pinolene) is a distillate from conifer resin and used as a film-forming antitranspirant that seals vulnerable tissues and curtails dehydration [18]. In viticulture, this terpene polymer decreased both foliar [19] and fruit [20,21] transpiration and resulted in altered fruit composition [22,23,24]. However, by physically blocking the stomatal pore, CO_2_ entry may also be reduced and thus net assimilation may decline. The objective of this work was to assess the effect of di-1-*p*-menthene on raspberry foliar gas exchange and fruit transpiration and to ascertain if improvements in water use efficiency may be achieved.

## 2. Materials and Methods

### 2.1. Plants

Biennial ‘’Tulameen’’ raspberry (*Rubus idaeus*, L.) plants at the 4–5 leaf stage were purchased from a local nursery, replanted in a premium garden mix in 30 × 40 cm pots and grown in a glasshouse at 25 °C day/18 °C night temperature. The plants were fertilized with one dose of slow release fertilizer (Osmocote, Scotts, Heerlen, Netherlands) and with a liquid fertilizer (MEGAMIX PLUS, Rutec, Tamworth, NSW, Australia consisting of 13% N, 10% P and 15% K) at the time of replanting and monthly thereafter. The pots were drip irrigated once daily to the point of run-off using 2.4 L/h pressure compensated drippers.

### 2.2. Leaf Surface Study

When the primocanes were approximately 1 m tall, leaves were chosen for treatment application based on node position, maturity and size. The compound leaves were composed of five leaflets. The third to fifth fully expanded leaves from the shoot apex, with an average central terminal leaflet lamina length of 10 cm, were marked with flagging tape and a 1% emulsion of Vapor Gard (Rawlinson & Brown, Griffith, NSW, Australia), and the active ingredient di-1-*p*-menthene was applied with a paintbrush to either the upper or lower surface of the center leaflet. The opposite side of these treated leaves was painted with dH_2_O. In the control treatment, dH_2_O was painted on the upper and lower surfaces. Each plant (*n* = 6) was given all three treatments simultaneously in order to minimize plant-to-plant variation in plant water status. The leaves were treated at midday, and measurements of leaf gas exchange were monitored at midday 24 h after application.

### 2.3. Light and Temperature Response of Individual Leaves to Infrared Gas Analyser Settings

Using different plants from the first study, the center leaflet of the youngest fully expanded leaves was dipped in (A) dH_2_O or (B) a 1% emulsion of di-1-*p*-menthene (*n* = 5 plants) until both sides of the leaflet were thoroughly covered. Leaf gas exchange was monitored 48 h after application.

### 2.4. Leaf Gas Exchange

Leaf net CO_2_ assimilation rate (*A*), stomatal conductance (*g*) and transpiration rate (*E*) were measured with a LI-6400 XT portable photosynthesis system (LiCor Biosciences, Lincoln, NE, USA) set to a reference CO_2_ concentration of 400 μmol CO_2_ mol^−1^. For the leaf surface study, the leaf chamber’s LED light source was set to a saturated light intensity of 1500 μmol m^−2^ s^−1^ and block temperature was set to 25 °C. For the light response study, the block temperature was set to 25 °C and PAR was reduced over ten intervals from 1500 to 0 µmol m^−2^ s^−1^ using the auto-program function. For the temperature response study, the light intensity was set to 1500 μmol m^−2^ s^−1^ and the block temperature was increased at 5 °C intervals from 25 to 40 °C. Measurements were made after light, temperature and CO_2_ readings were stable. Instantaneous transpiration efficiency (*A/E*) and intrinsic transpiration efficiency (*A/g*) were calculated from the measurements of *A*, *E* and *g*.

### 2.5. Fruit Transpiration

Fruit transpiration was measured by weighing detached berries over time. ‘’Cascade Delight’’ fruit were sampled from a commercial operation in Young, NSW. The plants were two years old; drip irrigated; and grown in deep, well-draining friable soil. The berries were excised randomly from one row of plants, taking one fruit per plant. The fruit were immediately sealed into plastic bags and transferred to the laboratory on ice, where within 2 h they were divided into five phenological stages (small green, medium green, green blush, white blush, and pink) and dipped in (A) dH_2_O or (B) a 1% emulsion of di-1-*p*-menthene (*n* = 6 berries per stage and treatment). Fruit were held at 20 °C and 60% RH and weighed at 4–6 h intervals over the subsequent 24 h. The average rate of weight loss was calculated using a linear regression. The fruit were subsequently dried at 60 °C until weights were stable. Berry water content was determined by subtracting dry weight from the fresh weight.

### 2.6. Statistical Analysis

Statistical analyses were performed with Genstat 18 statistical (VSN International Ltd., Hemel Hempstead, UK) software. For the leaf surface study, significant differences between treatments were determined by a one-factor ANOVA with treatment as a factor and plants as blocks. Least significant differences were calculated at 5% significance. For the light and temperature response studies, treatment differences were determined by a two-way anova with antitranspirant treatment and light or temperature as factors, and plants as blocks. Exponential curves (f = y0 + a ∗ (1 − exp(−b ∗ x)) + c ∗ (1 − exp(−d ∗ x))) were fitted to the light response data using Sigmaplot 14.0 graphing (Systat Software Inc., San Jose, CA, USA) software. For the fruit transpiration study, the linear regressions used to calculate average berry weight loss over time were highly significant with a correlation of 0.96 or greater. Treatment differences were determined by a 2-way anova with antitranspirant treatment and developmental stage as factors. All mean values are presented ± the standard error of the mean (s.e.m.) unless stated otherwise.

## 3. Results

**Figure 1:**
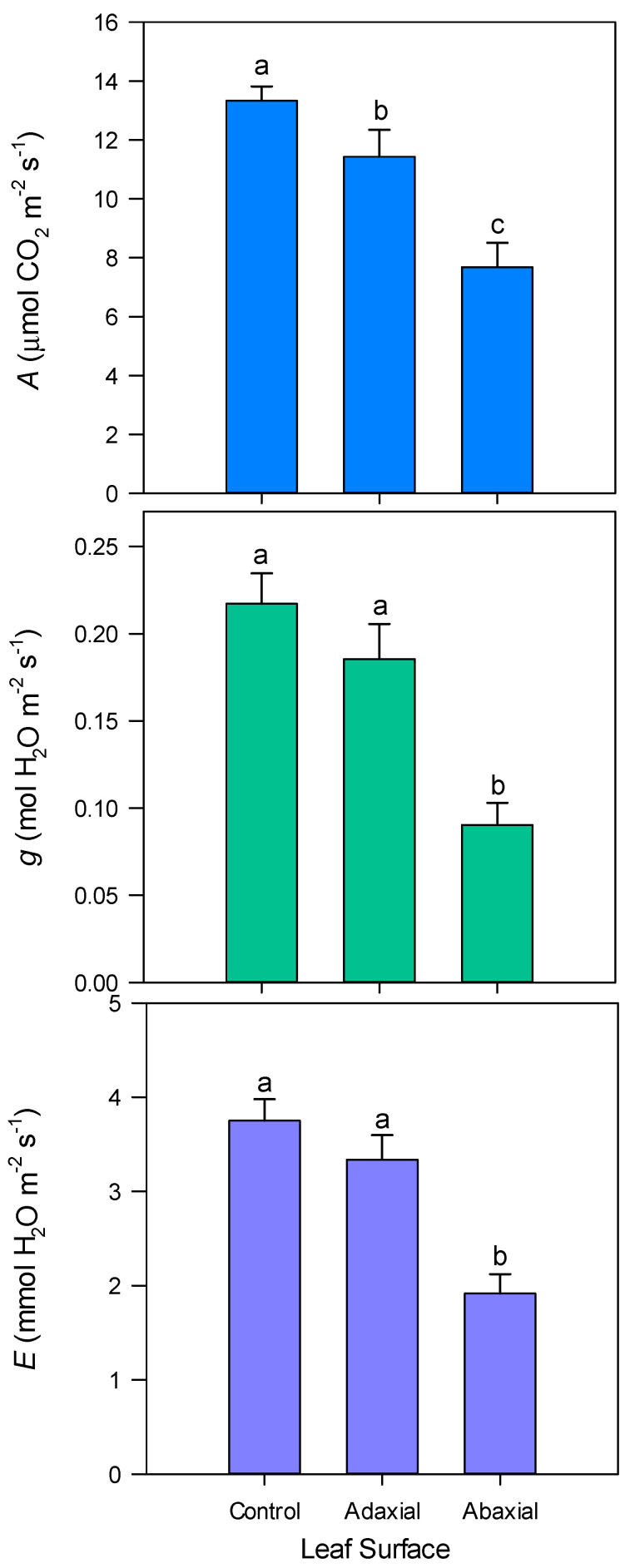
‘’Tulameen’’ raspberry leaf net assimilation (*A*), stomatal conductance (*g*) and transpiration (*E*) 24 h after treatment with 1% di-1-*p*-menthene on either the adaxial (upper) or abaxial (lower) leaf surface. Control leaves were treated with dH_2_O to both leaf surfaces. Measurements were carried out under light saturation and at 25 °C. Leaf gas exchange was monitored 24 h after application. Data are expressed as means ± s.e.m., *n* = 6, *p <* 0.001 for *A*, *g* and *E*. Different letters above the bars indicate statistically significant difference at *p* < 0.05.

Di-1-*p*-menthene was more effective when applied to the abaxial (lower) surface of the leaf compared to the adaxial (upper) surface (Figure 1). *A*, *g* and *E* were reduced by 42, 58 and 49 %, respectively, relative to untreated leaves (*p* < 0.001). Only *A* was significantly reduced, by 14%, when applied to the upper surface of the leaf.

**Figure 2:**
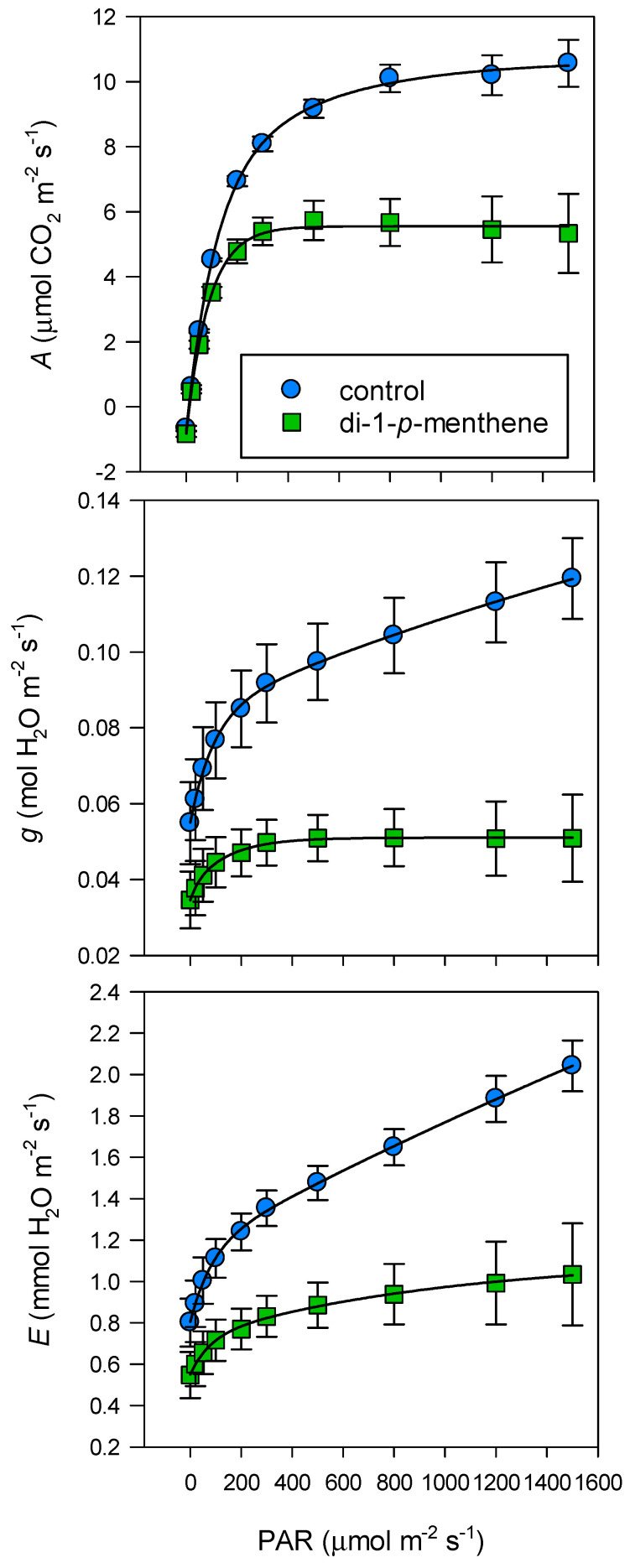
‘’Tulameen’’ raspberry leaf net assimilation (*A*), stomatal conductance (*g*) and transpiration (*E*) in response to a range of photosynthetically active radiation (PAR) following treatment with 1% di-1-*p*-menthene to both leaf surfaces. Control leaves were treated with dH_2_O only. Measurements were carried out at ambient CO_2_ and 25 °C. Leaf gas exchange was monitored 48 h after application. Data are expressed as means ± s.e.m., *n* = 5, *p* < 0.001 for *A*, *g* and *E*.

**Figure 3:**
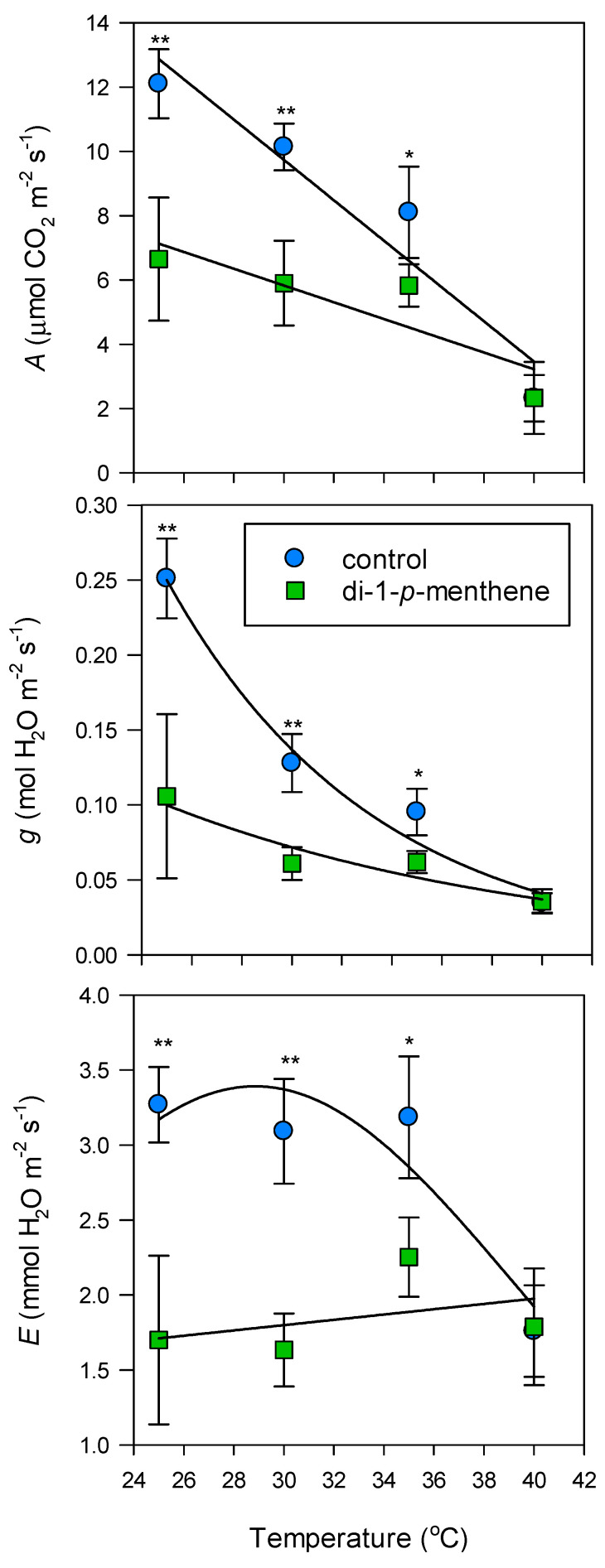
‘’Tulameen’’ raspberry leaf net assimilation (*A*), stomatal conductance (*g*) and transpiration (*E*) in response to temperature following treatment with 1% di-1-*p*-menthene. Control leaves were treated with dH_2_O only. Leaf gas exchange was monitored 48 h after application. The light intensity of the IRGA leaf chamber was set to 1500 μmol m^−2^ s^−1^ and the block temperature was increased at 5 °C intervals from 25 to 40 °C. Data are expressed as means ± s.e.m., *n* = 5, * *p* < 0.05, ** *p* < 0.01.

When applied to both surfaces, the reduction in gas exchange was evident across a wide range of PAR (*p* < 0.001) (Figure 2) and temperature (*p* < 0.001) (Figure 3). *A* of control leaves increased to 9.2 µmol CO_2_ m^−2^ s^−1^ at 500 PAR, but beyond this there were only slight increases (Figure 2). *A* of leaves treated with the antitranspirant increased to only 5.4 µmol CO_2_ m^−2^ s^−1^ at 300 PAR but *A* did not increase further with higher PAR. Stomatal conductance of control leaves increased to 0.12 mol H_2_O m^−2^ s^−1^ at 1500 PAR, but in treated leaves *g* only increased to 0.05 mol H_2_O m^−2^ s^−1^, without any further increases beyond 300 PAR. Similarly, *E* of control leaves increased to 2.0 mmol H_2_O m^−2^ s^−1^ at 1500 PAR but in treated leaves *E* increased to only 1.0 mmol H_2_O m^−2^ s^−1^, half that of the control.

Untreated leaves declined in *A* and *g* between 25 and 40 °C (Figure 3). The drop in *A* was linear, with 83% lower values at the highest temperature. Similarly, *g* declined by 80% from 0.25 to 0.05 mol H_2_O m^−2^ s^−1^. *E* did not alter between 25 and 35 °C, but at 40 °C it halved. The antitranspirant was effective at lowering *A*, *g* and *E* at 25, 30 and 35 °C; however, the effect diminished with increasing temperature due to the natural decline in these parameters. At 25 °C *A*, *g* and *E* were depressed by 45, 60 and 48 %, respectively. At 40 °C, the treatment was no longer significant due to very low levels that were apparent in the control leaves at this temperature.

**Figure 4:**
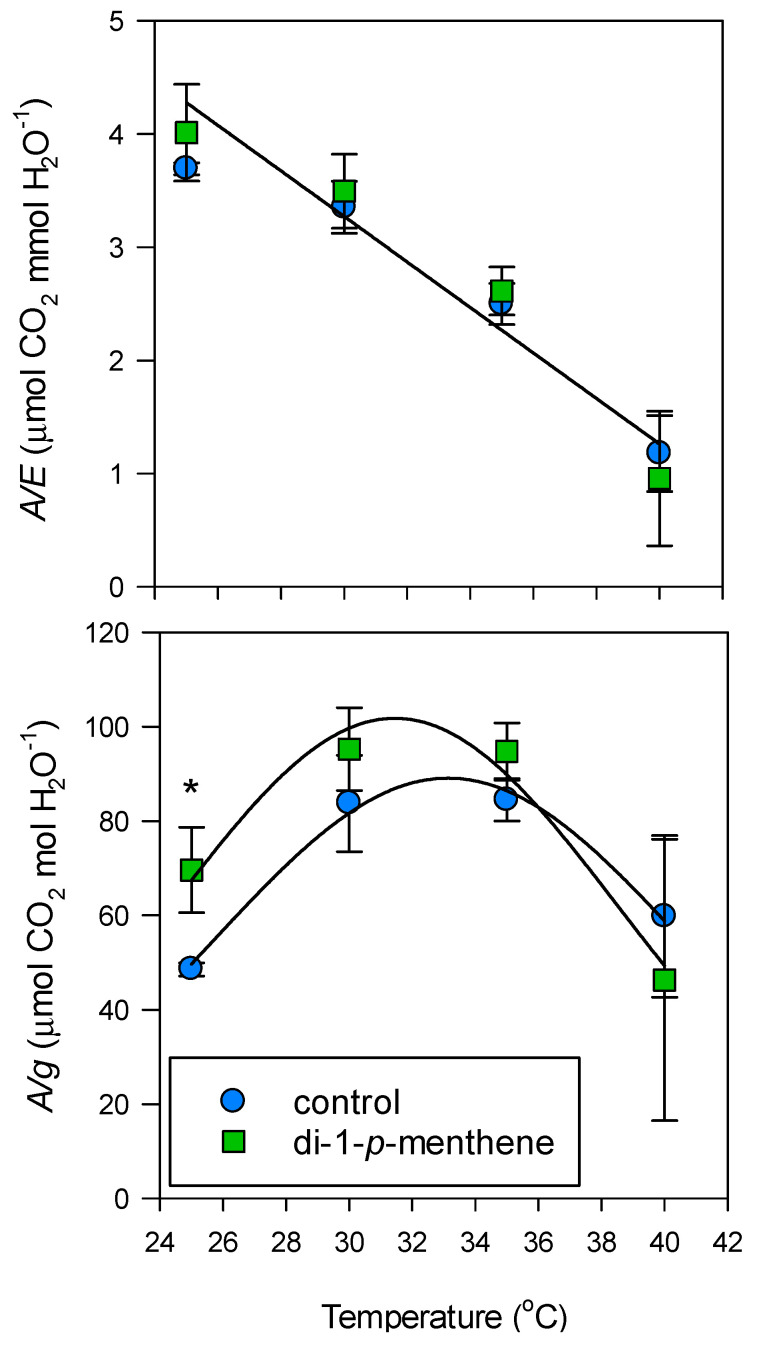
“Tulameen” raspberry instantaneous transpiration efficiency (*A/E*) and intrinsic transpiration efficiency (A/*g*) in response to temperature following treatment with 1% di-1-*p*-menthene. Control leaves were treated with dH_2_O only. Leaf gas exchange was monitored 48 h after application. The light intensity of the IRGA leaf chamber was set to 1500 μmol m^−2^ s^−1^, and the block temperature was increased at 5 °C intervals from 25 to 40 °C. Data are expressed as means ± s.e.m., *n* = 5, * *p* < 0.05.

Leaf *A/E* declined linearly between 25 and 40 °C and was not significantly improved (*p* = ns) with the application of the film at 25, 30, 35 or 40 °C (Figure 4). In contrast, as a consequence of the antitranspirant, leaf *A/g* was improved by 30% at 25 °C (*p* < 0.05). However, this was not consistent across the higher temperatures (Figure 4). *A/g* was two-fold greater at 30 and 35 °C for both control and treated leaves, but then declined again at 40 °C.

**Figure 5:**
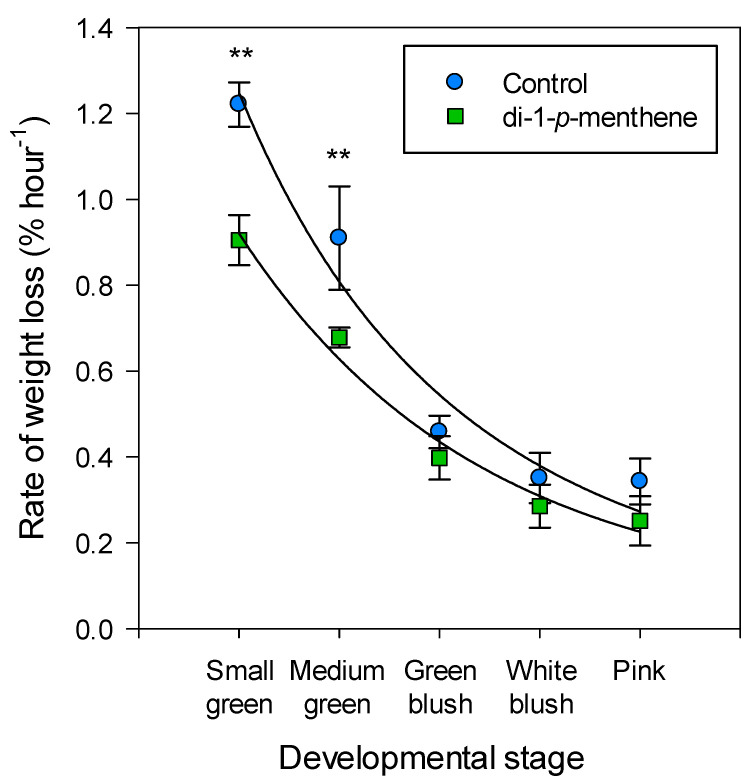
“Cascade Delight” fruit transpiration at the small-green, medium-green, green-blush, white-blush and pink developmental stages after treatment with dH_2_O or 1% di-1-*p*-menthene. Fruit were held at 20 °C and 60% RH and weighed at 4–6 h intervals over 24 h. Data are expressed as means ± s.e.m., *n* = 6, ** *p* < 0.01.

Di-1-*p*-menthene was effective at reducing fruit transpiration by 25% prior to the onset of ripening (Figure 5). However, with the onset of anthocyanin production and chlorophyll degradation, fruit at the green-blush and white-blush stages had lower transpiration rates naturally and the antitranspirant, at 1%, was not significantly effective at lowering this further.

## 4. Discussion

The antitranspirant di-1-*p*-menthene successfully suppressed raspberry leaf transpiration. This is in agreement with other horticultural crops such as grape [20,23,24], strawberry [25] and pepper [26]. We have shown here that the depression in transpiration is maintained across a 25 to 40 °C temperature range. This information is valuable in our warming world because water stress and heat often occur together; the elevated leaf-to-air vapour pressure deficit driven by extreme temperatures can result in plant water stress despite sufficient soil moisture, because root uptake is unable to keep up with the high evaporative demand [27]. The clear, flexible film was more effective on the lower leaf surface relative to the upper surface because in raspberries this is where most of the stomata are located [28].

Di-1-*p*-menthene reduced photosynthesis. This was expected because the film blocks CO_2_ more than H_2_O as a result of its greater molecular weight and similar polarity [15,29]. Other studies have correspondingly concluded that this antitranspirant reduces CO_2_ uptake by leaves [30]. The low light saturation point of film-covered leaves corroborates the hypothesis for limited diffusion of CO_2_ to the sites of carboxylation. This diffusive process was sensitive to temperature for both untreated and sprayed leaves of this temperate species, but especially so for the latter. Optimal temperature for photosynthesis in raspberry is around 17–21 °C [8], and this explains the linear decline in *A* we observed with each 5 °C increase in temperature. The depression in net carbon assimilation as a result of the antitranspirant suggests that vegetative and reproductive development may be prolonged or even incomplete in certain regions. However, this is quite speculative, and further work is required to substantiate this.

Despite reductions in photosynthesis, di-1-*p*-menthene was able to improve intrinsic water-use efficiency (*A/g*) at 25 °C but not at the higher temperatures. This is because *A/g* improved naturally in untreated plants at 30 and 35 °C, with the antitranspirant not able to bestow any further benefits. The decline in *A/g* at 40 °C in both treated and untreated plants is indicative of non-stomatal and biochemical limitations in photosynthesis. This is not an unusual consequence of heat stress; carboxylation capacity and the RuBP regeneration capacity directed by electron transport are both highly temperature dependent [31,32,33]. Di-1-*p*-menthene was not useful for improving *A/E* at any temperature, demonstrating that CO_2_ uptake was more severely hampered than H_2_O loss. The absence of improvements in water-use efficiency at the individual leaf level may suggest compromised yield, but this will need to be substantiated with assessments at the whole plant level. Yield was reduced in grape [19], was not affected in kiwi fruit [34] or pepper [35], and was improved in Sutlani fig [36]. These variable results underline the relevance of the entire integrated growing system and the specificities of when and how the antitranspirant is applied. We applied the antitranspirant at the 10-leaf stage, and further work should assess its impact if applied at later stages of development and at various levels of water stress.

The antitranspirant was effective not only at reducing the transpiration of leaves but also of fruit at the younger stages. Bunch transpiration was similarly reduced in grape, but it is noteworthy to mention that the effect was reduced as berries matured [20,37]. Lowered fruit transpiration as a result of film application on grapes in the field can result in bigger berries, because vascular flow into the fruit is maintained while water loss through the fruit’s surface is curtailed. This, however, may have negative consequences on fruit quality as a consequence of lower sugar concentrations through dilution [38]. Higher levels of the antitranspirant can be trialed in further studies; however, the carry-on impacts on fruit development and their sensory attributes will need to be assessed. In parallel, consumer resistance to chemical applications directly to the fruit will require careful consideration. Potential environmental effects will also need to be established, bearing in mind that other classes of antitranspirants can be detrimental to beneficial insects [39].

These preliminary tests form the basis for further work on whole plants and over a longer timescale. Canopy response may not necessarily mimic that of individual leaf response, and the complexities of VPD will need to be assessed under the intended growing system, either in completely enclosed, partially enclosed, or fully open systems. Large-scale applications over more than one season will allow growers to gain a more thorough understanding of the applicability of this antitranspirant to their specific growing conditions. The root environment, whether this may be limited to a container or unlimited in the field, will have an impact on plant water status and on stomatal responsiveness to the antitranspirant. The impact of the antitranspirant on ameliorating plant tissue damage or survival was not assessed, but if this is the objective it may be applied immediately preceding the extreme conditions so that the lower net assimilation does not have a long-lasting impact on fruit development in locations with short growing seasons. The duration of di-1-*p*-menthene efficacy requires further evaluation. Generally lasting for several weeks in the field [15] where foliage is exposed to sun and rain, it is likely that less frequent applications will be required in protected cropping scenarios. Other classes of natural antitranspirants may also be trialed, but the potential for reduced nutrient uptake as a consequence of the low transpiration will need to be considered. The application of compounds that upregulate the production of osmolytes to reduce osmotic stress or the production of antioxidants may offer further avenues to improve tolerance to water deficits.

## 5. Conclusions

Di-1-*p*-menthene reduced foliar transpiration and fruit transpiration at the young developmental stages. This antitranspirant may thus potentially be implemented as a tool to ameliorate desiccation at times when water is limited and/or evapotranspiration is extreme. Due to the predominance of abaxial stomata, the product will require application to the lower leaf surface for greatest effectiveness.
